# The prevalence of, and risk factors for, mycoplasma genitalium infection among infertile women in Ibadan: A cross-sectional study

**Published:** 2017-10

**Authors:** Tinuade Adesola Ajani, Timothy A. Olusesan Oluwasola, Mustapha A. Ajani, Rasheed Ajani Bakare

**Affiliations:** 1 *Department of Medical Microbiology, University College Hospital, Ibadan, Nigeria.*; 2 *Department of Obstetrics and Gynaecology, University College Hospital, Ibadan and College of Medicine, University of Ibadan, Ibadan. Nigeria.*; 3 *Department of Histopathology, Babcock University, Ilishan-Remo, Ogun State, Nigeria.*; 4 *Department of Medical Microbiology, College of Medicine, University of Ibadan, Ibadan, Nigeria.*

**Keywords:** Infertility, Mycoplasma genitalium, Prevalence, Risk factors, Screening

## Abstract

**Background:**

The association of mycoplasma genitalium (M. genitalium) with infertility has been documented. The infections are asymptomatic and difficult to diagnose. Understanding the associated risk factors will help in facilitating better screening measures for at-risk groups.

**Objective:**

The aim was to determine the prevalence of, and risk factors for, M. genitalium infection among infertile women in Ibadan.

**Materials and Methods:**

In this cross-sectional study, 402 women (267 infertile and 135 fertile) referred to 2 hospitals in Ibadan between March and November 2015 were enrolled. Information was obtained, using structured questionnaire, on sociodemographic and behavioral characteristics of the respondents while endocervical swabs were obtained for detection of M. genitalium. MgPa gene was diagnosed using the conventional Polymerase chain reaction. Bands corresponding to 495kb were documented as positive for M. genitalium.

**Results:**

Among the infertile women, 43 (16.1%) had evidence of M. genitalium infection as against 3 (2.2%) of women without infertility (p<0.001). Associated risk factors included having more than one lifetime sexual partner (OR=10.13, 95% CI: 3.76-33.97); husbands having other sexual partners (OR=12.88, 95% CI: 2.08-90.63); being a serial monogamist (OR=6, 95% CI: 4.35-8.27) and low socio-economic status (OR=2.80, 95% CI: 1.28-6.10). No relationship exists between the previous history of sexually transmitted infections and M. genitalium.

**Conclusion:**

The risk factors for M. genitalium infection are similar to those peculiar to other sexually transmitted infections. Its routine screening should be incorporated into the current protocol for microbiological evaluation of infertile women.

## Introduction

The current global infertility rate has been reported to be between 15-20% with the associated major economic burden on healthcare industry (1, 2). Mycoplasma genitalium (M. genitalium), an emerging sexually transmitted pathogen of the Mycoplasmatacea family, has been described as a major cause of various inflammatory conditions of the genital tract in women and non-gonococcal urethritis in men (3-5). The asymptomatic carrier state of M. genitalium is a major epidemiological concern as it enhances unnoticed transmission of the pathogen among sexual partners (6). In addition, challenges of culture and isolation of M. genitalium contribute to under-investigation, misdiagnosis and inadequate treatment of patients with the pathogen (7-9).

In Kenya, Cohen and colleagues reported the presence of M. genitalium in the cervix and/or endometrium of 16% of women with histological diagnosis of acute endometritis compared with 2% in women without endometritis (10). In a related study, Simms and co-workers linked M. genitalium to pelvic inflammatory disease (PID), where 13% of women with clinically diagnosed PID were M. genitalium positive on Polymerase chain reaction (PCR) compared with 0% in the control group. Similarly, another study in the United States by Haggerty and colleagues also reported a prevalence of 14% among patients with clinically diagnosed PID (11, 12). The evidences for M. genitalium cervicitis and PID suggested that the organism has potential to cause ascending infections thus resulting in infertility (11-14). Currently, in Nigeria, there is a dearth of studies on the overall prevalence and/or risk factors for acquiring M. genitalium in the general population and especially among patients with infertility. 

The main objective of this study was to determine the prevalence of M. genitalium among infertile women in our environment and the possible associated risk factors as this will contribute significantly to establishing preventive strategies among “at-risk” population and consequently reduce the burden of infertility in Nigeria.

## Materials and methods

This was a cross-sectional study involving 267 consenting women with a clinical diagnosis of infertility, and 135 women with proven fertility, conducted between March 1^st^, and November 30^th^, 2015. Using interviewer-administered questionnaire, we obtained information on socio-demographic characteristics and on risk factors associated with M. genitalium infection. 

Inclusion criteria were women within the reproductive age group, women with confirmed diagnosis of infertility irrespective of the type and women who were attending the postnatal clinic which served as a proof of recent delivery of live babies. The women were also willing to allow endocervical samples to be taken for diagnosis of M. genitalium infection. On the other hand, the exclusion criteria were women who have been diagnosed to have sexually transmitted infections (STIs) or PIDs, those who were on antibiotics or who used antibiotics within the previous 6 months and also those who were not willing to provide consent. 

Ethical standards were followed in the handling, storage, and disposal of specimens. Using sterile Copan eNat cervical swabs, endocervical swabs were collected under aseptic condition for each recruited woman, preserved with eNat preservation medium and stored at -20^o^C until processing. Jona Bioscience Bacteria DNA preparation kit was used for DNA extraction in accordance with the manufacturer’s instruction. The extracted DNA was amplified by conventional polymerase chain reaction, PCR (Eppendorf thermal cycler; Nexus series).

Primer sequences used for M. genitalium Mgpa (major adhesion protein) gene primer were Mgp-F 5ʹAAG TGG AGC GAT CAT CAT TAC TAA C-3ʹ and Mgp-R 5ʹ CCG TGG TTA TCA TAC CTT CTG A- 3ʹ. PCR set up comprised of 5 µl of DNA extract, 0.40 µl of primers (forward and reverse), 10.60 µl of PCR water and 4µl of Master mix (reaction buffer B, MgCl2, DNTP, blue and yellow dye) all in a tube. All tubes were sealed and briefly centrifuged before amplification in PCR machine. After amplification, electrophoretic separation of PCR products was performed on 1.5% agarose gel stained with ethidium bromide, and visualized by Ultraviolet illumination (15). 

**Figure 1:**
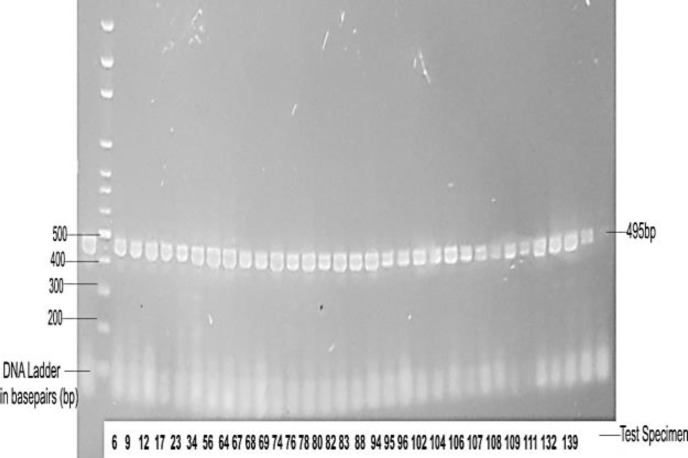
Gel electrophoresis of PCR amplicons for Mycoplasma genitalium

About 10 μl of DNA ladder at 100bp gradient was placed at one end of wells of 1.5% agarose gel stained with 2μl ethidium bromide. Thereafter, 20 μl of DNA extract was added to each well on the agarose gel, placed into the electrophoretic tank and switched on at 100 volts for 60 min. The tray containing the agarose gel and bands were transferred to a bio-imaging system and results were read on a computer (Figure 1).

Bands corresponding to 495kb on the DNA ladder were documented as positive for M-genitalium and those that did not meet the criteria were recorded as negative.

**Ethical consideration**

Written informed consent was obtained from eligible participants. Ethical approval was obtained from the Joint Ethics Committee of the University of Ibadan and University College Hospital Ibadan before commencement (UI/EC/12/0201). 

**Statistical analysis**

Descriptive and inferential statistical analysis of the data was done using the Statistical Package for the Social Sciences, version 20.0, (SPSS Inc, Chicago, Illinois, USA). Variables of interest included levels of education and income, past history of infection with Neisseria gonorrhea or any other STIs, personal or partner’s use of male barrier contraceptive device (condom), the current number of sexual partners, number of lifetime sex partners and number of alcohol intake per day. Means and standard deviations were derived for quantitative variables while proportions were derived for qualitative variables. Association between categorical variables was determined using Chi-square test at statistical significance level of set 5%. Relationship between multiple variables were further determined using the multivariate logistic regression.

## Results

Our study was aimed at determining the prevalence of, and associated risk factors for M. genitalium in Ibadan. It was a cross-sectional, hospital-based study involving 267 infertile women with mean age of 33.8±5.7 yr out of which majority, 156 (58.4%), had secondary infertility. In addition, 135 women with proven fertility were also recruited to serve as controls and their mean age is similar to that of the infertile group. Most of the respondents were married (95.1%), were Christians (62.2%) and had a tertiary level of education (58.1%) (Table I). Overall, M. genitalium infection was positive in 43 (16.1%) of the infertile group and 3 (2.2%) of the controls. There was a slightly higher prevalence of M. genitalium among those with secondary infertility 27 (17.3%) as against 16 (14.4%) of those with primary infertility although the difference is not statistically significant.

The relationship between commonly identified factors and acquisition of M. genitalium infection is described in table II. Family type especially serial monogamy, monthly income less than the National minimum wage of 18,000 naira (equivalent of 50 US Dollars) implying low socioeconomic status, daily alcohol consumption and having multiple sexual partners (having more than one-lifetime sex partners and/or have husbands who have other sex partners) are significantly associated with acquiring the pathogen. About two-thirds, 62.5%, of participants whose partners have other sexual partners have a positive result for M. genitalium infection. However, there was no association between M. genitalium and age, past history of Neisseria gonorrhea and other STIs and the use of condoms. 

On logistic regression analysis of factors that are independently significant for M. genitalium infection, it was discovered that being single, being a serial monogamist, having spouses with more than one sexual partners and having more than one lifetime sexual partners remained significant (Table III). Participants whose husbands have other sexual partners are almost 13 times at risk of acquiring M. genitalium infection (OR=12.88, 95% CI: 2.08-90.63) while those respondents who have more than one-lifetime sexual partner are ten times at risk of the infection (OR=10.13, 95% CI: 3.76-33.97). In addition, being a serial monogamist increases the risk by 6 (OR=6.0, 95% CI: 4.35-8.27) and those with low socioeconomic status have about triple the risk of infection (OR=2.80, 95% CI: 1.28-6.10).

**Table I:** Socio-demographic factors of the participants with infertility

**Variables**		**n (%)**
Age Group (yr)		
	20-24	9 (3.4)
	25-29	52 (19.5)
	30-34	90 (33.7)
	35-39	71 (26.6)
	≥ 40	45 (16.9)
Marital Status		
	Married	254 (95.1)
	Single	12 (4.5)
	Divorced	1 (0.4)
Education		
	Primary	19 (7.1)
	Secondary	93 (34.8)
	Tertiary	155 (58.1)
Religion		
	Christianity	166 (62.2)
	Islam	101 (37.8)
Type of infertility:		
	Primary	111 (41.6)
	Secondary	156 (58.4)
Previous history of abortion:		
	Yes	118 (44.2)
	No	149 (55.8)

* Descriptive analysis showing frequency and percentages

**Table II:** Factors associated with Mycoplasma genitalium

**Variable **		**Mycoplasma genitalium**		**χ** ^2^	**p-value**
		**Yes**	**No**		
Grouped Age ( yr)				3.3	0.190
	20-29	14 (23.0)	47 (77.0)		
	30-39	21 (13.0)	140 (87.0)		
	≥40	8 (17.8)	37 (82.2)		
Family type				8.3	0.040
	Single	9 (19.6)	37 (80.4)		
	Monogamous	31 (16.7)	155 (83.3)		
	Polygamous	2 (5.9)	32 (94.1)		
	Serial monogamist	1 (100.0)	0 (0.0)		
Monthly income (in naira)				8.3	0.004
	<18,000	15 (29.4)	36 (70.6)		
	≥18,000	28 (13.0)	188 (87.0)		
Past history of Gonorrhoea				3.7	0.159
	Yes	19 (19.0)	81 (81.0)		
	No	19 (12.8)	130 (87.2)		
	Don’t know	5 (27.8)	13 (72.2)		
Use of condom				2.1	0.143
	Yes	9 (24.3)	28 (75.7)		
	No	34 (14.8)	196 (85.2)		
Husband has other sex partners				14.3	0.001
	Certainly yes	5 (62.5)	3 (37.5)		
	Certainly no	27 (16.6)	136 (83.4)		
	Not sure	11 (11.5)	85 (88.5)		
Lifetime sexual partners				29.9	<0.001
	One	5 (3.8)	128 (96.2)		
	>One	38 (28.4)	96 (71.6)		
Daily alcohol consumption (in bottles)				9.0	0.011
	None	38 (15.0)	215 (85.0)		
	1	4 (57.1)	3 (42.9)		
	>1	1 (14.3)	6 (85.7)		

Data presented as n (%).

* Chi-square test statistics were used and p<0.05 was accepted as statistically significant

**Table III:** Logistic regression analysis

**Variables**		**Odds Ratio**	**95% CI**	**p-value**
Family type		Monogamous (Reference)		
	Single	1.22	0.49-2.95	0.64
	Polygamous	0.31	0.05-1.44	0.105
	Serial monogamist	6.0	4.35-8.27	0.027
Monthly income, in naira (dollar)				
	≥18,000 (50)-Reference			
	<18,000 (50)	2.80	1.28-6.10	0.004
Husband has other sex partners				
	Certainly no (Reference)			
	Certainly yes	12.88	2.08-90.63	0.00012
Lifetime sexual partners				
	One (Reference)			
	>One	10.13	3.76-33.97	<0.0001

* Odds ratio was used to explain the logistic regression analysis and p<0.05 was accepted as statistically significant

## Discussion

Our study, which was aimed at identifying risk factors that are associated with the acquisition of M. genitalium among infertile women in Ibadan, Southwest Nigeria, found a prevalence of 16.1% among patients with infertility and 2.2% among women without infertility. This difference is statistically significant and implied that M. genitalium is associated with infertility. Previous studies from Nigeria had reported a prevalence of 4% among asymptomatic, infertile males in South-southern part of Nigeria and 6% among asymptomatic adolescents in South-eastern Nigeria but no studies had been done among infertile women to the best of our knowledge (14, 16).

In comparison with similar studies in other environments, our prevalence is slightly higher than that of Grześko and his colleagues but lower than that of some other researchers (4, 17-19). The observed variations may be due to differences in the assay method employed as most studies using serological assays are limited in sensitivities because of cross-reactions between the antibodies of M. genitalium and M. pneumonia. However, Rajkumar and his colleagues, using real-time PCR, also obtained higher prevalence rate in a pilot population although with patients who have primary infertility having a much higher rate than those with secondary infertility (20). Real-time PCR has been suggested to have more detection sensitivity than conventional PCR thereby implying that, in the absence of racial differences, the prevalence rate in our environment may actually be higher than we obtained (21-23).

The risk factors that are confirmed to be associated with M. genitalium infection from this study included: family type (single and serial monogamy) and multiple sexual partners in which the respondents have more than one lifetime sex partners and/or have husbands who equally have other sexual partners thus corroborating the findings of previous studies (4, 24-28). Thurman and his co-workers reported that M. genitalium was detected in 9.5% and 10.6% of women and men in heterosexual relationships reporting to an STI clinic (27). However, these subjects were five times more likely to be infected with M. genitalium if their sexual partner was M. genitalium positive. Similarly, Tosh and his colleagues also reported that despite a high prevalence rate of 13.6% for M. genitalium among 383 young women, only one of the respondents who tested positive for the pathogen was sexually inexperienced (28). The increase in the probability of acquisition of infection among participants whose husbands have other sexual partners was likely driven by its association with sexual activity. This therefore confirms the plausible suggestion that transmission requires repeated exposure to an infected partner–which is often facilitated by the longer-term partnerships that are characteristic of cohabiting individuals.

It is interesting to note that being single is associated with the acquisition of M. genitalium although this is similar to the report from a study among sex-workers in China (29). Diverse reasons have been advanced for this finding ranging from cultural reasons which shift the blame of infertility on just one partner to that of some ladies opting to be single mothers in order to avoid the troubles and skirmishes of marriage. However, our study was unable to establish any association between past infection with Neisseria gonorrhea or any other STI and M. genitalium. This result is similar to that of Hancock *et al* but in contrast with that from Vandepitte and co-workers in a study conducted among commercial sex workers (30, 31). 

However, the lack of association between the previous infection with Neisseria gonorrhea or other STIs and M. genitalium in this study may be due to factors such as recall bias, deliberate under-reporting or asymptomatic infections. Meanwhile, the lack of association between condom use and M. genitalium in our study is consistent with previous studies in Africa (31, 32). This is understandable as our study participants were being managed for infertility and as such were not expected to be using barrier contraceptive methods with their partners.

## Conclusion

In summary, the prevalence of M. genitalium is significantly higher among infertile patients and may be an important potential pathogen for female infertility among our study population. It is important to note that the prevalence rate may still be higher than obtained if there are opportunities to use real time PCR. The risk factors found to be associated with M. genitalium infection lend out further support for the sexual transmissibility of M. genitalium while being unmarried and having multiple sexual partners have been shown to be independent risk factors for its acquisition. Understanding these risk factors will enable targeted efforts at their prevention in order to reduce the burden of infertility in Nigeria. While infections like genital tuberculosis have been proven to have no relationship with unexplained infertility, it is apparent that further studies are required to determine the extent of involvement of M. genitalium in the cause and course of infertility among our women (33).
